# Seizure Prophylaxis in Patients with Traumatic Brain Injury: A Single-Center Study

**DOI:** 10.7759/cureus.753

**Published:** 2016-08-26

**Authors:** Shannon Inglet, Margaret Baldwin, Amie H Quinones, Sarah Majercik, Dave S Collingridge, Joel MacDonald

**Affiliations:** 1 Department of Pharmacy, Intermountain Medical Center; 2 Department of Trauma and Surgical Critical Care, Intermountain Medical Center; 3 Office of Research, Intermountain Medical Center; 4 Neurosurgery, University of Utah

**Keywords:** seizure, prophylaxis, traumatic brain injury, outcome

## Abstract

The use of prophylactic anticonvulsants to prevent early post-traumatic seizures (PTSs) is recommended but inconsistently employed in patients with traumatic brain injury (TBI). The authors evaluated outcomes associated with prophylaxis administration in patients with TBI at a Level 1 trauma center. All patients admitted with TBI from October 2007 through May 2012 were included. Our primary outcome was the incidence of early PTSs. Secondary outcomes included mortality, length of hospital and intensive care unit (ICU) stays, and incidence of late seizures. Of the 2,111 patients with TBI, 557 (26.4%) received seizure prophylaxis and 1,554 (73.6%) did not. Two early PTSs occurred in the prophylaxis group (0.4%), whereas 21 occurred in the non-prophylaxis group (1.4%) (p = 0.05). The overall mortality rate was higher in patients who received prophylaxis (14.2% vs. 6.2%; p < 0.001), and the mean hospital length of stay (LOS) was longer (6.8 ± 6.9 vs. 3.8 ± 5 days; p < 0.001). In patients with severe and moderate TBI, the rate of prophylaxis administration was approximately half, whereas significantly fewer patients with mild TBI received prophylaxis than did not (20.2% vs 79.8%, p < 0.001). Lower Glasgow Coma Scale (GCS) score and longer hospital LOS were associated with early PTS (p = 0.008 for both comparisons), but sex and age were not. Brain hemorrhage was present in 78.3% of those patients who experienced early seizures. In our cohort, patients who received seizure prophylaxis had a lower GCS score, higher overall mortality rate, longer LOS, and more frequent ICU admissions, suggesting that patients who received prophylaxis were likely more severely injured.

## Introduction

Approximately 1.7 million traumatic brain injuries (TBIs) occur in the US each year, and they result in about 52,000 deaths [1]. TBI is associated with substantial disability, socioeconomic burden, and mortality [1-3]. Individuals who suffer a TBI are at higher risk for seizures because of both focal and diffuse brain tissue damage [4-5]. Post-traumatic seizures (PTSs) are classified as early if they occur within seven days of injury or late if they occur beyond seven days [2-3, 5]. Currently, the Brain Trauma Foundation guidelines recommend the use of prophylactic anticonvulsant medications to prevent early PTSs, even though early PTSs are not associated with worse outcomes [2, 6-8]. This recommendation is based on conflicting results from studies that were based on the use of phenytoin and valproic acid [6-9]. As a result, seizure prophylaxis for TBI patients remains controversial. The purpose of this study was to evaluate outcomes associated with prophylaxis administration in patients with TBI at a single Level 1 trauma center.

## Materials and methods

This retrospective study was approved by the Intermountain Medical Center Institutional Review Board (approval #1040256). Informed patient consent was obtained at the time of treatment. We used the hospital system’s enterprise data warehouse (EDW) and trauma registry database (TraumaBase 7, Clinical Data Management, Genessee, Colorado) to identify all patients with acute TBI that were admitted to a single Level 1 trauma center from October 1, 2007, through May 31, 2012. Patients with TBI were identified based on ICD-9 diagnosis codes 850–854. Patients were excluded if they were younger than 14 years old, had a history of a pre-injury seizure disorder, or underwent a decompressive craniotomy.

The primary outcome measure was the incidence of early PTS; we compared the incidence among the group of patients who received seizure prophylaxis with the incidence among those who did not. PTS was also identified via ICD-9 codes. Prophylactic anti-seizure medications included phenytoin, fosphenytoin, valproic acid, phenobarbital, and levetiracetam. Other medications that are known to lower the seizure threshold (e.g., antipsychotics and benzodiazepines) were also tabulated to control for potential confounders. Secondary outcomes included the difference in mortality, hospital length of stay (LOS), intensive care unit (ICU) LOS, and incidence of late seizures between the two groups. Age, sex, and Glasgow Coma Scale (GCS) score on admission were also compared. TBI severity was categorized according to admission GCS score as severe (3–8), moderate (9–12), or mild (13–15).

### Statistical analysis

Statistical analyses were performed using IBM SPSS, version 19 (IBM, Armonk, NY). Discrete variables were analyzed using Fisher’s exact or Chi-square tests. Continuous data were analyzed using a student t-test and Wilcoxon-rank sum test, as appropriate. Hierarchical binary logistic regression was used to assess whether relevant independent variables influenced the occurrence of seizures. Covariates were entered first, and those not reaching significance were removed one by one from the regression analysis. The main predictor variable, prophylaxis receipt, was entered last. This approach enabled us to assess the independent contribution of prophylaxis on the primary outcome. Statistical significance was set a priori at 0.05.

## Results

**Figure 1:**
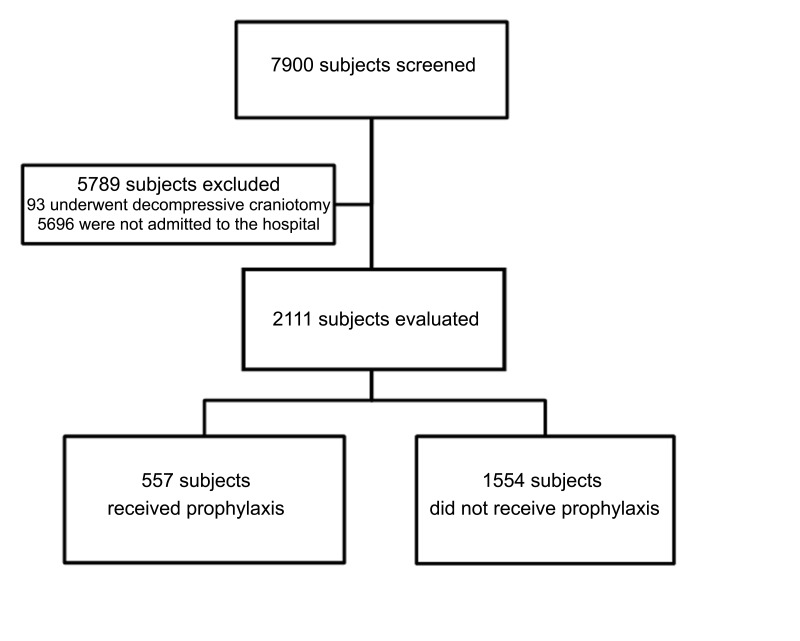
Flowchart indicating patient selection

During the study period, 7,900 patients over 14 years of age without a seizure disorder presented to our emergency department with a TBI. Of these, 5,696 were not admitted to the hospital, and 93 patients underwent decompressive craniotomy for severe brain injury; these patients were excluded from the study. The remaining 2,111 patients were included in the analysis (Figure 1). Demographic data for the study cohort are presented in Table 1.

**Table 1:** Demographic Data of Patients Without a Seizure Disorder Who Were Admitted With a TBI TBI = traumatic brain injury ^a ^p-value for comparison between prophylaxis and no-prophylaxis groups NS: not significant

Characteristic	Total Population	Treated with Prophylaxis	No Seizure Prophylaxis	P-value^a^
Median age (years)	49	56	46.5	p < 0.001
Percentage of male patients	64.4	66.1	63.8	NS
Percentage of patients presenting with GCS score				
3 – 8	15.5	29.2	10.9	p < 0.001
9 – 12	3.9	7.5	2.7	P < 0.001
13 – 15	80.6	63.3	86.5	p < 0.001
Percentage of patients with each mechanism of injury				
Fall	47.4	59.3	43.2	
Motor vehicle accident	19.1	16.2	20.2	
Motorcycle accident	5.8	4.7	6.2	
Bicycle accident	3.8	3.2	4.1	
Pedestrian accident	4.6	3.8	5	
Assault	10	5.2	11.8	
Other	9.1	7.7	9.6	

### Primary outcome

Seizure prophylaxis was given to 557 (26.4%) patients during their hospital admission and not given to 1,554 (73.6%). Twenty-three (1.1%) patients experienced early PTS. Two of these patients received prophylaxis (0.4%), while 21 patients received no prophylaxis (1.4%) (p = 0.05).

### Secondary outcomes

Hierarchical binary logistic regression was performed to account for the influence of GCS, hospital LOS, sex, and age while evaluating the influence of prophylaxis on PTS. These results showed that low GCS score and longer hospital LOS were associated with early PTS (p = 0.008, OR = 0.89, and p = 0.008, OR = 1.06, respectively). Receipt of prophylaxis reduced the likelihood of early PTS (p = 0.014, OR = 0.149). Age and sex had no predictive effect on early PTS. There were two late seizures in the group receiving prophylaxis (0.4%) and four in the group receiving no prophylaxis (0.3%) (p = 0.66). Among the patients with severe TBI, seizures occurred in 0.7% of those who received prophylaxis and 4.4% of those who did not (p = 0.06). Although this difference was not statistically significant, it may be clinically relevant.

**Figure 2:**
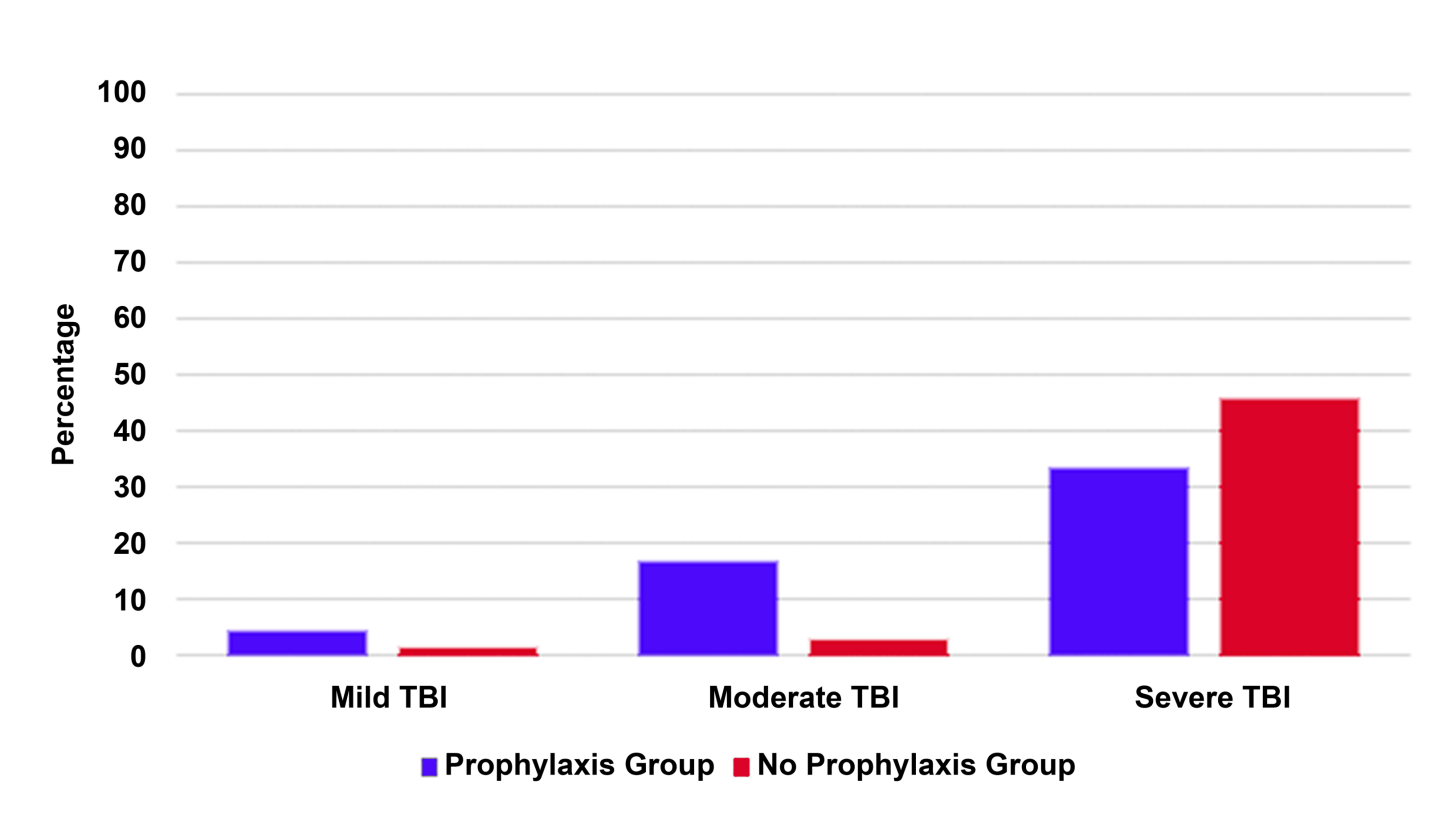
Graph showing rates of ICU admission and mortality for patients with TBI

Patients who received prophylaxis had an overall mortality rate of 14.2% compared with 6.2% for patients who received no prophylaxis (p < 0.001) (Figure 2). Among patients with severe TBI, mortality was lower in those who received prophylaxis compared with those who did not (32.9% vs 45.6%, p = 0.026).

**Figure 3:**
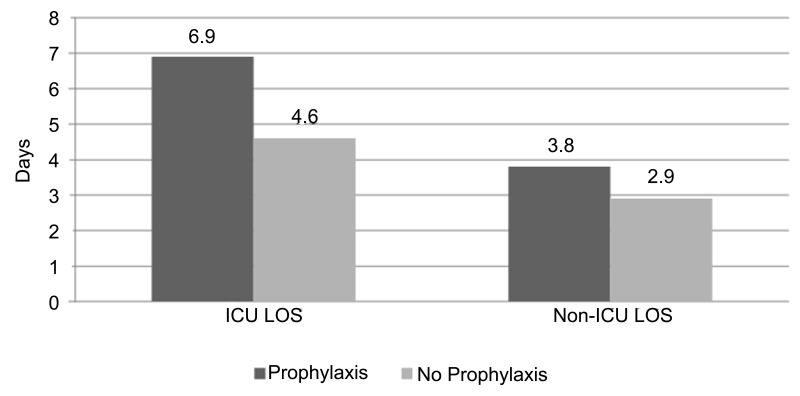
Graph illustrating length of stay in the hospital and in the ICU for patients with TBI

Ninety-five percent of patients who received seizure prophylaxis were admitted to the ICU from the emergency department, whereas 51% of patients who received no prophylaxis were admitted (p < 0.001). Of those patients admitted to the ICU, the patients who received prophylaxis had a mean ICU LOS of 4.5 ± 5.6 days, whereas the mean ICU LOS in the patients who did not receive prophylaxis was 2.9 ± 4.9 days (p < 0.001). Hospital LOS was also longer in the patients who received prophylaxis than in those who did not (6.8 ± 6.9 days vs. 3.8 ± 5 days, p < 0.001) (Figure 3).

### Additional analyses

**Figure 4:**
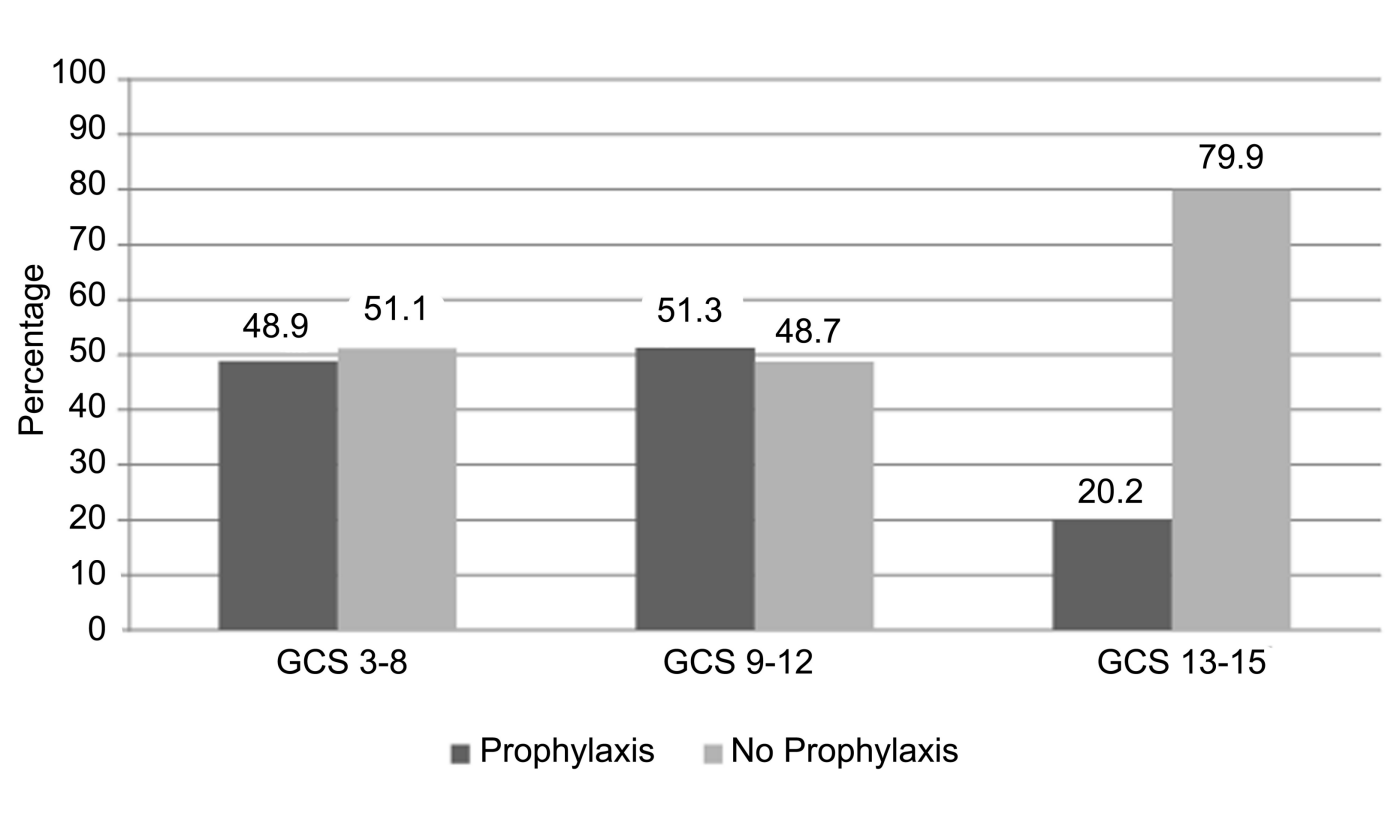
Graph demonstrating rates of prophylaxis administration to patients with TBI by Glasgow Coma Scale score

Among patients who experienced seizures, 78.3% had visible intracranial blood on the admission CT scan. Subdural hematoma was present in 27.8% of cases, intraparenchymal hemorrhage was present in 11.1%, and a combination of bleed type was present in half of all seizure cases. Patients in the prophylaxis group were older than those in the non-prophylaxis group (average age: 56 years vs. 46.5 years, p < 0.001). Prophylaxis administration did not differ by sex: 25.8% of females and 27.7% of males were given prophylaxis. Mild TBI (as assessed by GCS) accounted for 80.6% of all TBI, whereas 3.9% were moderate and 15.5% were severe. Of all patients with either severe or moderate TBI, similar proportions received prophylaxis versus no prophylaxis (48.8% vs 51.2%, p = 0.73, and 51.3% vs 48.7%, p = 0.91, respectively). In contrast, fewer patients with mild TBI received prophylaxis than did not (20.2% vs 79.8%, p < 0.001) (Figure 4).

## Discussion

Our results suggest that there is a statistically significant difference in the incidence of early PTS among TBI patients who received seizure prophylaxis and those who did not (p = 0.05). Early PTSs were rare in our cohort and occurred in only 1.1% of all study subjects. Among patients with severe TBI, seizures tended to be less likely in those who received prophylaxis, although this difference was not statistically significant.

Patients who received seizure prophylaxis were older, had lower GCS scores on admission, and were more often admitted to the ICU. These patients also had a higher overall mortality rate and longer LOS when compared with those patients who did not receive seizure prophylaxis. These results suggest that patients who received prophylaxis were more seriously injured. The fact that GCS and hospital LOS were independent predictors of early PTS also supports this finding. In a subgroup of patients with severe TBI, we found that mortality was actually lower in those who received prophylaxis, which may be indicative of some protective effect.

Previous authors have shown that patients with severe TBI have a higher incidence of PTS [4, 10]. Temkin, et al. [6] found a statistically significant difference in the incidence of early PTS in patients with severe TBI treated with phenytoin compared with patients treated with a placebo (3.6% versus 14.2%). Conversely, Young, et al. found no statistically significant difference between groups in a similar study of patients with severe TBI [8]. Our study included all patients diagnosed with TBI across the spectrum of injury severity. Overall, our cohort demonstrated a much lower seizure incidence than was observed in previously published studies. A recent study published by Inaba, et al. similarly evaluated patients with all severities of TBI and found a seizure incidence of 1.5%, which compares favorably with our findings [11].

There are several potential limitations associated with this study. First, because the study is retrospective in nature and uses data from a single center, the veracity of the data relies on the quality of information abstraction and entry. Our institution, as an American College of Surgeons–verified Level I Trauma Center, maintains a trauma registry and is also compelled by state law to record data for every trauma patient in a statewide central trauma registry. The data management process at our institution is the responsibility of a few well-trained individuals and, therefore, may actually be more reliable than other reports.

We used ICD-9 codes to identify patients with TBI for inclusion. This may have introduced errors because of the inherent imprecision of the ICD-9 coding schema. It is possible that we inadvertently included patients who did not fit our inclusion criteria. Since our study group was fairly large, it is unlikely that this type of error would influence our results. We attempted to mitigate this potential error by cross-referencing our EDW-derived list of patients with records in our institution’s trauma registry.

Finally, the study may have been underpowered to detect a difference in the primary outcome since the incidence of PTS is very low. It is possible that a larger cohort could show a more significant difference in the incidence of early PTS among TBI patients who received prophylaxis and those who did not.

Despite these limitations, we believe that our findings are both reliable and of clinical value. We concede that larger controlled trials would be needed to clarify this issue. Our results suggest that the incidence of early PTS at our institution is low and that the administration of seizure prophylaxis does not appear to be useful for the majority of TBI patients. Nonetheless, its selective administration to patients who have severe TBI and are at an increased risk of mortality may still offer some benefit.

## Conclusions

We found a significant difference in the incidence of early PTS between those patients who received seizure prophylaxis and those who did not. When patients were stratified by TBI severity, mortality was lower in patients with severe TBI who received prophylaxis compared with those who did not. This finding may identify a group of patients in whom prophylaxis may be beneficial. Intracranial hemorrhage of any magnitude appears to be associated with seizure (78.3%). Overall, patients who received seizure prophylaxis tended to be older, stayed in the hospital longer, and were more likely to die than those who did not. Seizure prophylaxis may be preferentially administered to sicker patients.
